# Cytochrome *c* in cancer therapy and prognosis

**DOI:** 10.1042/BSR20222171

**Published:** 2022-12-22

**Authors:** João Pessoa

**Affiliations:** CNC - Center for Neuroscience and Cell Biology, CIBB - Center for Innovative Biomedicine and Biotechnology, University of Coimbra, Coimbra, Portugal

**Keywords:** apoptosis, cancer, cytochrome c, prognosis, stability, therapy

## Abstract

Cytochrome *c* (cyt *c*) is an electron transporter of the mitochondrial respiratory chain. Upon permeabilization of the mitochondrial outer membrane, cyt *c* is released into the cytoplasm, where it triggers the intrinsic pathway of apoptosis. Cytoplasmic cyt *c* can further reach the bloodstream. Apoptosis inhibition is one of the hallmarks of cancer and its induction in tumors is a widely used therapeutic approach. Apoptosis inhibition and induction correlate with decreased and increased serum levels of cyt *c*, respectively. The quantification of cyt *c* in the serum is useful in the monitoring of patient response to chemotherapy, with potential prognosis value. Several highly sensitive biosensors have been developed for the quantification of cyt *c* levels in human serum. Moreover, the delivery of exogenous cyt *c* to the cytoplasm of cancer cells is an effective approach for inducing their apoptosis. Similarly, several protein-based and nanoparticle-based systems have been developed for the therapeutic delivery of cyt *c* to cancer cells. As such, cyt *c* is a human protein with promising value in cancer prognosis and therapy. In addition, its thermal stability can be extended through PEGylation and ionic liquid storage. These processes could contribute to enhancing its therapeutic exploitation in clinical facilities with limited refrigeration conditions. Here, I discuss these research lines and how their timely conjunction can advance cancer therapy and prognosis.

## Introduction

Effective cancer treatment depends not only on therapeutic approaches with high specificity to malignant cells but also on accurate prognosis markers. Attaining these two critical goals demands the detailed characterization of the biochemical alterations of cancer cells. Those alterations can uncover molecules whose concentrations, modifications, and/or subcellular locations are altered. Their return to normal status during cancer therapy can be monitored to assess therapeutic efficacy. Consequently, correcting the status of a molecule with altered concentration, modification, and/or subcellular location in cancer can have a therapeutic outcome. As discussed below, recent research has shown that cytochrome *c* (cyt *c*) is a strong candidate for a prognosis marker and for a therapeutic agent.

Cytochromes were first described and named by David Keilin in 1925 [1]. He found four absorption bands in the absorption spectra of yeast cells and tissues from animals and plants. He acknowledged similar spectroscopic observations by C. A. MacMunn, published between 1884 and 1887, but received with skepticism. From those four absorption bands, Keilin identified a ‘cellular pigment’, which he named ‘cytochrome’. He proposed it to be composed of three compounds, identified as *a*′, *b*′, and *c*′ [1]. Their roles in the mitochondrial respiratory chain have been assigned. Cytochrome *b* is one of the subunits of coenzyme Q-cyt *c* reductase (complex III) and cytochrome *a* is one of the subunits of cyt *c* oxidase (complex IV) [2]. Cyt *c* is a monomeric protein located in the mitochondrial intermembrane space, between complexes III and IV.

Over the decades, extensive research on cyt *c* has contributed critical insight not only into mitochondrial respiration (reviewed in [3]) but also into apoptosis [4,5], a form of programmed cell death. Upon proapoptotic stimuli, the mitochondrial outer membrane is permeabilized, triggering the efflux of cyt *c* from the mitochondrial intermembrane space into the cytoplasm [6]. In the cytoplasm, cyt *c* binds to apoptotic protease-activating factor-1 (Apaf-1) [7], resulting in a sequence of biochemical reactions causing the activation of caspases, a class of proteases that execute apoptosis by degrading cellular components [8,9]. Apoptosis is a process of the highest relevance in cancer. First, it is generally inhibited in cancer cells [10]. Second, the induction of cancer cell apoptosis has outstanding therapeutic potential [11]. Mitochondrial outer membrane permeabilization is considered a point of no return in the triggering of the intrinsic pathway of apoptosis [12]. One of its direct consequences is the efflux of cyt *c* from the mitochondrial intermembrane space into the cytoplasm. Decreased levels of cyt *c* have been detected in cancer tissues, suggesting apoptosis inhibition. For example, cyt *c* levels were decreased in glioma tissues in relation to healthy ones, and further decreased in more advanced disease stages [13]. Accordingly, cyt *c* overexpression inhibited tumor growth in a mouse model of clear cell renal cell carcinoma [14]. On the other hand, the knockdown of cyt *c* in a clear cell renal cell carcinoma cell line increased its proliferation, likely through the inhibition of apoptosis. Cyt *c* overexpression in these cells increased apoptosis rates [14]. Additionally, several widely utilized chemotherapeutic drugs induce apoptosis by promoting the efflux of cyt *c* into the cytoplasm (reviewed in [15]). Therefore, the increase in cytoplasmic cyt *c* levels correlates with the capacity of combatting the disease.

A recent review [15] discusses the biochemical properties of cyt *c* that enable its repurposing as a proapoptotic chemotherapeutic drug and describes several cyt *c* cell delivery systems for cancer therapy. The present article aims at complementing that study. Here, I discuss additional aspects of cyt *c* as a molecular tool in cancer. (1) Serum cyt *c* as a biomarker for cancer prognosis. (2) Biosensors for cyt *c* quantification in human serum, highlighting their detection sensitivities. (3) Additional details of the cyt *c* therapeutic delivery systems, including model systems in which they have been tested and their inhibitory concentrations. (4) The impact of cyt *c* point mutations, post-translational modifications, and redox state in its proapoptotic function. (5) Approaches for increasing cyt *c* thermal stability, to facilitate its storage and therapeutic utilization under limited refrigeration conditions. Finally, I discuss further perspectives, including the potential of cyt *c* in the prognosis of other diseases.

## Serum cyt *c* in cancer prognosis

Cytoplasmic cyt *c* can evade the cell. Consistently, in breast cancer tumor samples, cyt *c* was released from epithelial cells into the lumen of the cancerous duct [16]. Due to its cell evasion ability, cyt *c* can reach the bloodstream. Therefore, its quantification in the serum can reflect its cytoplasmic levels.

Decreased serum levels of cyt *c* have been detected in cancer. In newly diagnosed non-small lung cell cancer patients, serum cyt *c* levels were about three-fold lower than in healthy individuals [17]. Cyt *c* levels were also decreased in the serum of clear cell renal cell carcinoma patients [14]. These observations are consistent with a lack of apoptosis induction in cancer due to insufficient cyt *c* levels in the cytoplasm. Nevertheless, different cancer patients can have different levels of serum cyt *c*. Although there was no evident correlation between serum levels of cyt *c* and the tumor differentiation stage, clear cell renal cell carcinoma patients with higher cyt *c* levels had higher survival rates than those with lower levels of the protein [14]. In patients with other cancer types, increased serum levels of cyt *c* were also correlated with an increased probability of patient survival [18]. Although elevated levels of serum cyt *c* can also indicate increased tumor content [18], their increase during cancer therapy has been related to more favorable prognoses [14].

In addition to its correlation with favorable prognosis, high serum levels of cyt *c* can also reflect increased apoptosis levels induced by cancer therapy [18]. In non-small lung cell cancer patients, serum cyt *c* increased at least 13-fold after the first cycle of chemotherapy [17]. Chemotherapy could also raise serum cyt *c* levels in patients with hematologic malignancies, including acute myeloid leukemia and non-Hodgkin lymphoma [19]. Non-small lung cell cancer patients with relatively higher cyt *c* levels before chemotherapy had a higher cyt *c* increase upon this treatment [17]. These observations indicate that chemotherapy resulted in increased cyt *c* levels in the patient serum and increased levels of tumor apoptosis. Chemotherapeutic drugs were able to induce mitochondrial outer membrane permeabilization and cyt *c* efflux into the cytoplasm and furthermore to the extracellular medium, thus increasing serum levels of cyt *c*. In adult T-cell leukemia patients, the levels of serum cyt *c* were proposed to be more sensitive than those of lactate dehydrogenase to track changes in tumor status [19]. The rapid response of cytoplasmic cyt *c* levels to apoptosis-inducing chemotherapy makes this protein a convenient marker to assess the outcome of cancer therapies [20].

**Figure 1:**
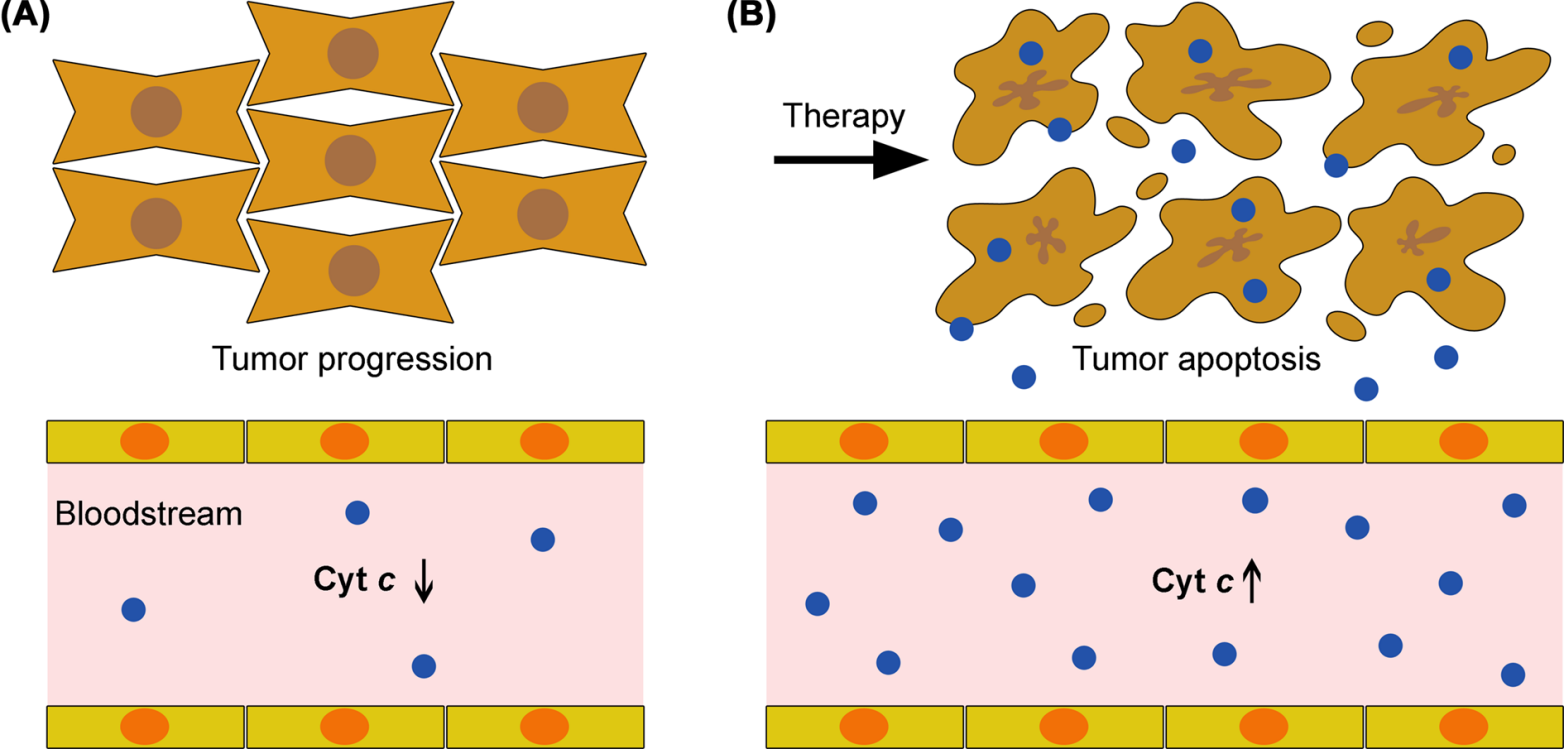
Serum cyt *c* in cancer prognosis (**A**) Apoptosis is inhibited in tumors, correlating with decreased cyt *c* levels in the bloodstream. (**B**) The therapeutic induction of apoptosis in tumors results in increased levels of cytoplasmic cyt *c*, which can further reach the bloodstream. The detection of increased cyt *c* levels in the serum is correlated with a favorable prognosis.

These studies show that quantification of cyt *c* in patient serum is useful to monitor the patient’s response to chemotherapy. Decreased serum levels of cyt *c* can indicate apoptosis inhibition and tumor progression (Figure 1A). On the other hand, proapoptotic cancer therapy is generally able to increase cyt *c* levels in the serum (Figure 1B). These studies anticipate serum cyt *c* as a noninvasive prognostic marker in cancer. In these studies, cyt *c* was quantified using immunologic approaches, including enzyme-linked immunosorbent assay (ELISA) [17,18], immunohistochemistry [14], Western blotting [19], and electrochemiluminescence immunoassays [20]. The relevance of cyt *c* quantification in patient serum has encouraged the development of innovative approaches for highly sensitive cyt *c* quantification in biological samples, which will be described in the following section.

## Biosensors for quantification of serum cyt *c*

**Figure 2:**
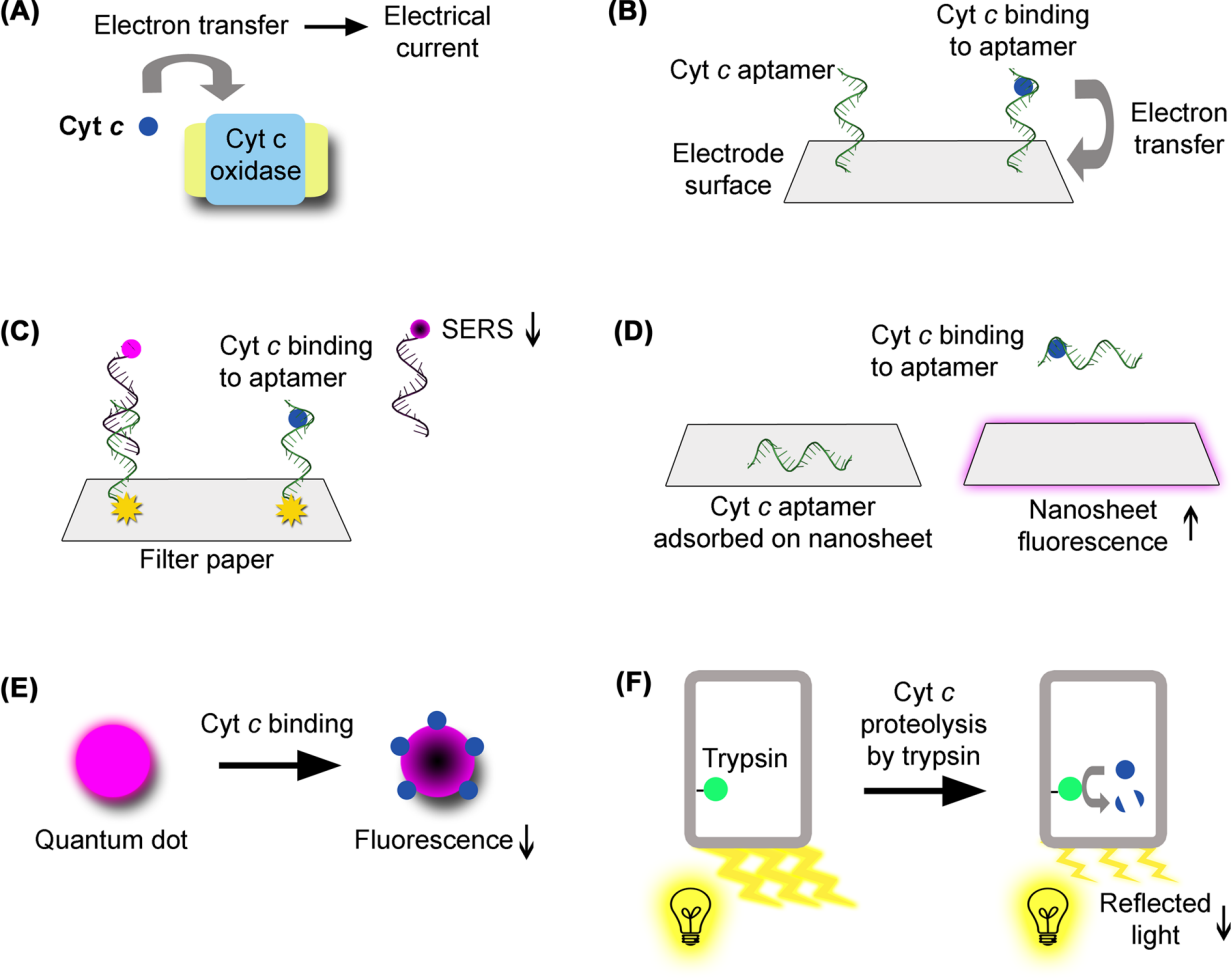
Biosensors for cyt *c* quantification in human serum (**A**) Cyt *c* (represented in blue) can be detected in the serum through its electron transfer to cyt *c* oxidase, which generates a measurable electrical current. (**B**) Cyt *c* can also be detected through the binding to a specific aptamer (represented in green) immobilized on an electrode surface. Electron transfer from cyt *c* to the electrode generates an electrical signal. (**C**) In a SERS biosensor, a cyt *c* aptamer is attached to a gold nanoparticle (represented in yellow) immobilized on filter paper. The aptamer is also hybridized with a fluorescently labeled complementary oligonucleotide (represented in black). Cyt *c* binding to the aptamer results in its dissociation from the oligonucleotide, whose physical separation from the gold nanoparticle decreases its SERS signal. (**D**) In a fluorescent biosensor, a cyt *c* aptamer is adsorbed on a nanosheet, quenching its fluorescence. Cyt *c* binding to the aptamer induces its dissociation from the nanosheet, which becomes fluorescent. (**E**) Quantum dots are intrinsically fluorescent. Upon cyt *c* binding, their fluorescence is quenched. (**F**) Cyt *c* can also be detected through its proteolysis by trypsin (represented in green) immobilized inside nanoporous structures. Cyt *c* proteolysis generates a reaction product that decreases the intensity of the light reflected by the porous nanostructures. Elements were not drawn to scale. For simplicity, some of the elements of these biosensors were omitted. In electrochemical biosensors, reference and auxiliary electrodes are not represented.

There has been an extensive development of biosensors for quantification of cyt *c* in human serum. Currently, ELISA [17,18] is likely the most used method. Nevertheless, this review is focused on more recent and innovative technical approaches for the same purpose (Table 1). These novel biosensors are antibody-free and exploit diverse detection methods. In some of these systems, cyt *c* is recognized through its electrical properties. Some biosensors are based on cyt *c* oxidase associated to electrodes, in which electron transfer from cyt *c* to cyt *c* oxidase generates or affects electrical currents [21,22] (Figure 2A). In another electrochemical biosensor, cyt *c* fitting into specific recognition nanocavities lowers the electrical current established between an electrolyte solution and a gold electrode [23]. In other approaches, the specific binding of cyt *c* to an aptamer [24,25] has been used for detection based on differential pulse voltammetry [26], surface-enhanced Raman scattering (SERS) [27], and fluorescence [28]. In the differential pulse voltammetry biosensor, the cyt *c* aptamer is immobilized on the surface of a glassy carbon electrode containing carbon nanofibers. When cyt *c* binds to the aptamer, its heme transfers electrons to the electrode, generating an electrical signal [26] (Figure 2B). In the SERS biosensor, the cyt *c* aptamer is hybridized with a fluorescently labeled oligonucleotide attached to a gold nanoparticle immobilized on a filter paper surface. Cyt *c* binding to the aptamer induces its dissociation from the oligonucleotide, decreasing the intensity of the SERS signal, due to increased distance to the gold nanoparticle [27] (Figure 2C). In the fluorescence-based biosensor, the aptamer is adsorbed on the surface of a graphitic carbon nitride nanosheet, whose fluorescence is quenched. Upon cyt *c* binding, the aptamer dissociates from the nanosheet, enhancing its florescence [28] (Figure 2D). Other biosensors utilize quantum dots [29], whose fluorescence is quenched upon cyt *c* binding [30,31] (Figure 2E). One of these systems is sensitive to trypsin activity. Cyt *c* proteolysis by trypsin restores the quantum dot fluorescence decreased upon cyt *c* binding [30]. In the other biosensor, the fluorescence decrease associated to cyt *c* binding is more pronounced at higher temperatures [31]. There is one additional biosensor in which trypsin is immobilized inside porous nanostructures. Cyt *c* detection results in its proteolysis by trypsin and the consequent generation of a reaction product that decreases the intensity of reflected light [32] (Figure 2F). These systems exploit specific features of cyt *c*, such as its binding to an aptamer or the electrical currents generated from electron transfer originating in its heme.

**Table 1:** Biosensors for quantification of cyt *c* in human serum

Reference	Year of publication	Detection method	Biosensor description	Sensing mechanism	Additional features	Detection range (μg/ml)^1^	Detection limit (μg/ml)^1^
[21]	2007	Square wave voltammetry	Cyt *c* oxidase immobilized in didodecyldimethylammonium bromide micelles associated to gold electrodes	The presence of cyt *c* affects the electrical current resulting of electron transfer between cyt *c* oxidase and gold electrodes	Contains a platinum wire auxiliary electrode and a silver/silver chloride reference electrode	2.3–118	2.3 (in human serum)
[22]	2020	Electrochemistry	Cyt *c* oxidase immobilized on graphene oxide nanoparticles deposited on a pencil graphite electrode	Electron transfer from cyt *c* to cyt *c* oxidase generates an electrical current through graphene oxide nanoparticles to the pencil graphite electrode	Contains a platinum wire auxiliary electrode and a silver/silver chloride reference electrode	0.04–0.18	0.04 (in solution)
[23]	2022	Differencial pulse voltammetry	Gold disk electrode coated with a polyortophenylenediamine polymer containing cyt *c* recognition cavities immersed into a ferrocenecarboxylic acid solution	Cyt *c* fitting inside its recognition cavities lowers the electrical current generated by electron transfer from ferrocenecarboxylic acid to the gold electrode	Contains a platinum wire counter electrode and a silver/silver chloride/potassium chloride reference electrode. Polyortophenylenediamine provides electrical isolation	1.3 × 10^−6^–4.1 × 10^−5^	4.2 × 10^−8^ (in diluted human serum)
[26]	2022	Differencial pulse voltammetry	Cyt *c*-binding aptamer immobilized on the surface of a glassy carbon electrode modified with carbon nanofibers	Cyt *c* binding to aptamer induces electron transfer from cyt *c* to the electrode, generating an electrical signal	Aptamer immobilization is enhanced through the modification of the electrode surface with graphene oxide functionalized with aspartate. Carbon nanofibers enhance the electrical conductivity of the glassy carbon electrode. Also contains a silver/silver chloride/potassium chloride reference electrode and a platinum wire auxiliary electrode	0.1175–1175	8.7 × 10^−3^ (in healthy human serum)
[27]	2020	SERS	Cyt *c* aptamer hybridized with an oligonucleotide labeled with cyanine-5 attached to gold nanoparticles immobilized on a filter paper	Cyt *c* binding to aptamer dissociates its complementary oligonucleotide; SERS signal of the oligonucleotide decreases due to its increased distance to the gold nanoparticle	Gold nanoparticles have sharp protrusions to enhance their magnetic fields	1.79 × 10^−6^–10	1.79 × 10^−6^ (in serum from non-small cell lung cancer patients)
[28]	2017	Fluorescence	Cyt *c*-binding aptamer adsorbed on the surface of a graphitic carbon nitride nanosheet	Nanosheet fluorescence is quenched through aptamer adsorption. Upon cyt *c* binding, the aptamer changes its structure and dissociates from the nanosheet, enhancing nanosheet fluorescence	Detection range and detection limit are compared with those from other biosensors	0.19–1.65	0.031 (in spiked human serum)
[30]	2019	Fluorescence	Titanium carbide quantum dots functionalized with protective ε-poly-L-lysine	Cyt *c* binding to quantum dots quenches their fluorescence	Also detects trypsin activity; cyt *c* proteolysis by trypsin restores fluorescence	2–470	2 × 10^−3^ (in solution)
[31]	2021	Fluorescence	Nitrogen and fluorine codoped carbon dots	Cyt *c* binding to carbon dots quenches their fluorescence	Temperature-sensitive: temperature increase decreases fluorescence intensity	6–294	2.9 (in solution)
[32]	2020	Interferometric reflectance spectroscopy	Nanoporous anodic alumina pore walls functionalized with trypsin via a 3-aminopropyl trimethoxy silane linker	Trypsin digestion of cyt *c* generates a reaction product that decreases the intensity of reflected light	Light intensity decreases as a logarithmic function of cyt *c* concentration	0.01–1	0.006 (in solution)

1 When originally indicated in the μM range, the values of these parameters were converted into μg/ml through multiplication by the cyt *c* molecular weight (11.749 kDa).

Additional biosensors have also been developed for quantification of cyt *c* in cell lysates [33–39], in nonserum solutions, and/or visualization in living cells [34,40–46]; however, these studies do not include validation for detection of cyt *c* in human serum. Nevertheless, they prove the possibility of designing and building a vast array of highly sensitive cyt *c* detection systems.

## Systems for therapeutic delivery of cyt *c* to cancer cells

Cyt *c* is amenable to biotechnological applications in cancer not only through its quantification in human serum but also through its therapeutic delivery to cancer cells. The capacity of cyt *c* to induce the apoptosis of cancer cells has inspired the development of a diverse set of molecular tools for its delivery and release into the cytoplasm of cancer cells. Many of these systems have been summarized in a recent review [15]. The cyt *c* delivery systems can be generally divided into fusion protein-based systems and nanoparticle-based systems. To avoid widespread cytotoxicity, many of them contain a protein or peptide that recognizes a membrane protein specifically expressed on the surface of cancer cells. This modification promotes selective recognition and endocytosis of the cyt *c* delivery system by these cells. To minimize any possible cytotoxic effects, endocyted exogenous substances can be rapidly transferred to lysosomes, resulting in their lysosomal degradation. This protective mechanism may create an obstacle to the therapeutic action of an endocyted drug. Therefore, while testing novel drugs, it is convenient to assess if they are able to evade from endosomes and escape lysosomal degradation. Upon endocytosis, many of these cyt *c* delivery systems were shown to efficiently evade the endosomes and avoid lysosomal degradation (Tables 2–5), which would otherwise require higher doses for therapeutic efficacy. In the cytoplasm of cancer cells, cyt *c* release induces apoptosis. In pharmacology, the IC_50_ parameter corresponds to the drug concentration that induces 50% of its maximum response. Here, IC_50_ is the cyt *c* concentration needed to reduce cancer cell viability in about 50%.

**Table 2:** Fusion protein-based cyt *c* therapeutic delivery systems

Reference	Year of publication	Fusion protein description	Additional features	Endosomal/lysosomal escape assessed	Approximate IC_50_ (μg/ml)^1^	Cancer type(s)	Model systems used (except negative controls)
[47]	2021	Three tandem cyt *c* molecules fused to an anti-human epidermal growth factor receptor 2 (HER-2) single-chain antibody	Increasing the number of cyt *c* molecules increased anticancer effectiveness. Lacks a cleavable linker	No	8.3	HER-2-positive breast cancer	SK-BR-3 and BT-474 (HER-2-positive) and MDA-MB-231 and MCF-7 (HER-2-negative); SK-BR-3 cells xenograft mouse model
[48]	2018	Cyt *c* fused to transferrin via a disulfide bond	The disulfide bond is cleaved under reductive cytoplasmic environment, releasing cyt *c*. Nontoxic to MRC5 healthy lung cells	Yes	4000	Cancer types overexpressing the transferrin receptor	A549 lung cancer cells, HeLa cervical cancer cells, and K562 chronic myleloid leukemia cells
[49]	2020	Cyt *c* fused to chlorotoxin via an enterokinase cleavage site	Amenable to purification of chlorotoxin	Yes	300–400	Glioma; cancer types expressing the chlorotoxin receptor	9L/lacz and NIH/3T3 cells
[50]	2014	Cyt *c* fused to galactosylated albumin via a reducible disulfide bond	Albumin is a substrate for modification with galactose, the ligand recognized by the asialoglycoprotein receptor on cancer cells. The disulfide bond is cleaved under reductive cytoplasmic environment, releasing cyt *c*	Yes	(Not estimated)	Asialoglycoprotein receptor-positive hepatocellular carcinoma (was also internalized by hepatocellular carcinoma cells lacking this receptor)	HepG2, Hep3B, and Mahlavu cells

1 The IC_50_ values originally listed in μM were converted into μg/ml through multiplication by the molecular weight (in kDa) of the respective fusion protein.

**Table 3:** Nanoparticle-based therapeutic delivery systems where cyt *c* is encapsulated inside molecular pockets

Reference	Year of publication	Nanoparticle description	Additional features	Endosomal/lysosomal escape assessed	IC_50_ (μg/ml)	Cancer type(s)	Model systems used (except negative controls)
[51]	2019	Cyt *c* encapsulated inside humanized *Archaeoglobus fulgidus* ferritin	Recognizes cells expressing CD71 on their surface	No	(Not estimated)	Acute promyelocytic leukemia	NB4 cells
[52]	2020	Cyt *c* encapsulated inside polypeptide-based micelles	For hypoxic tumors. Hypoxia promotes cyt *c* release, which is slower under normoxia	Yes	50 (under hypoxia)	Hepatocellular carcinoma	HepG2 cells
[53]	2016	Cyt *c* encapsulated inside a hyaluronic acid-based nanogel containing disulfide bonds whose cleavage disrupts the nanogel and releases cyt *c*	Intrinsically fluorescent. Hyaluronic acid recognizes CD44 on the cell surface	No	36.1 (MCF-7 cells)^1^	Breast cancer	MCF-7 and U87 cells; MCF-7 cells xenograft mouse model
[54]	2021	Cyt *c* encapsulated inside a polyglycerol-based nanogel containing cystamine-derived linkers, whose cleavage under reductive conditions releases cyt *c*	80% of encapsulated cyt *c* was released within 48 h	No	30	Lung, cervical, and breast cancer	A549, HeLa, and MCF-7 cells

1 The IC_50_ value originally indicated as 3.07 μM was converted into μg/ml through multiplication by the cyt *c* molecular weight of 11.749 kDa.

**Table 4:** Therapeutic delivery systems where cyt *c* is co-precipitated into nanoparticles

Reference	Year of publication	Nanoparticle description	Additional features	Endosomal/lysosomal escape assessed	IC_50_ (μg/ml)	Cancer type(s)	Model systems used (except negative controls)
[55]	2014	Cyt *c* precipitated into nanoparticles, further modified with a poly(lactic-co-glycolic) acid polymer. Under a reductive cellular environment, the linker connecting the polymer to the protein is cleaved via its disulfide bond, releasing cyt *c*	Induced apoptosis in HeLa cells more efficiently that solo cyt *c*	Yes	50	Cervical cancer	HeLa cells
[56]	2020	Cyt *c* precipitated into nanoparticles further decorated with folate-poly(ethylene glycol)-poly(lactic-co-glycolic acid)-thiol. In the cytoplasm, reductive environment cleaves the disulfide bond between cyt *c* and the co-polymer, releasing cyt *c*	Recognizes cancer cells expressing the folate receptor	Yes	49.2–70.1	Cervical cancer and Lewis lung carcinoma	HeLa cells; Lewis lung carcinoma cells and mouse model
[57]	2022	Cyt *c* precipitated into nanoparticles stabilized by reversible cross-linking with dithiobis(succinimidyl propionate), containing reducible disulfide bonds	Decorated with a folic acid-polyethylene glycol polymer, to recognize cancer cells expressing the folate receptor; optimized version of [56]	Yes	47.46	Cervical cancer and Lewis lung carcinoma	HeLa cells; Lewis lung carcinoma cells and mouse model
[58]	2012	Nanoparticles composed of cyt *c* conjugated with a membrane-permeable peptide, apolipoprotein A1, and the 1,2-dioleoyl-3- trimethylammonium-propane and dioleoylphosphatidylethanolamine lipids	Further modified with anisamide, a ligand that recognizes the sigma receptor on H460 lung carcinoma cells; has shown decreased liver uptake in mice	Yes	(Not estimated)	Non-small cell lung cancer	H460 cells; H460 cells xenograft mouse model
[59]	2017	Nanoparticle complex of cyt *c* and cardiolipin; cardiolipin increases membrane permeability	Apoptosis was further stimulated through the production of lipoperoxide radicals by the nanoparticle; could induce apoptosis in doxorubicin-resistant cells	No	270 ± 60; 460 ± 30	Ovarian carcinoma	A2780 and A2780-Adr cells

**Table 5:** Therapeutic delivery systems where cyt *c* is bound to the surface or encapsulated inside nanoparticle pores

Reference	Year of publication	Nanoparticle description	Additional features	Endosomal/lysosomal escape assessed	Approximate IC_50_ (μg/ml)	Cancer type(s)	Model systems used (except negative controls)
[60]	2018	Cyt *c* chemically bound to the surface of iron oxide nanoparticles containing a gold coating	Conjunction with cyt *c* nanoparticles enhanced the cytotoxic effects of doxorubicin, paclitaxel, oxaliplatin, vinblastine, and vincristine	Yes	(Estimated in conjunction with chemotherapeutic drugs; widely variable)	Hepatocellular carcinoma	HepG2, Huh-7D, and SK-hep-1 cells
[61]	2013	Cyt *c* modified with sulfosuccinimidyl6-[3′-(2-pyridyldithio)- propionamido] hexanoate and bound to mesoporous silica nanoparticles via redox-sensitive disulfide bonds. In the cytoplasm, disulfide bonds are cleaved and cyt *c* is released	Four lactose molecules were bound to each cyt *c*, to prevent its degradation	Yes	40–50	Cervical cancer	HeLa cells
[62]	2014	Cyt *c* and doxorubicin encapsulated inside enlarged pores of mesoporous silica nanoparticles sealed with linkers containing disulfide bonds. Cyt *c* was modified with aptamers recognizing nucleolin (a membrane marker of tumorigenesis and angiogenesis). In the cytoplasm, reductive environment cleaves the disulfide bonds and releases doxorubicin, cyt *c*, and the aptamer	Triple effect: doxorubicin blocks DNA replication, cyt *c* induces apoptosis, and the aptamer destabilizes DNA repair	Yes	(Not estimated)	Hepatocellular carcinoma	HepG2 cells; HepG2 xenograft mouse model
[63]	2021	Cyt *c* encapsulated inside enlarged pores of mesoporous silica nanoparticles. Amino-terminated polyethylene glycol (PEG)-modified gold nanoparticles seal the pores through electrostatic interactions, preventing cyt *c* release. Inside lysosomes, acidic pH reverses their electrical charge, causing the detachment of gold nanoparticles and the release of cyt *c*	pH-sensitive; cyt *c* is released at pH 5.0	Yes	15.8	Cervical cancer	HeLa cells; HeLa cells xenograft mouse model
[64]	2020	Positively charged cyt *c* loaded into enlarged negatively charged pores of mesoporous silica nanoparticles, through electrostatic interactions. Under low pH, nanoparticle charge becomes positive and releases cyt *c*	pH-sensitive; cyt *c* is released at pH 5.0. Nanoparticle roughness increased cellular uptake and apoptosis	No	20	Ovarian cancer	SKOV3 cells
[65]	2010	Cyt *c* and a fluorescent dye encapsulated inside the cavities of hyperbranched polyhydroxyl polymer nanoparticles coated with folate. Cyt *c* was attached through electrostatic interactions between lysine amino acids and hydroxyl groups of the polymer	Recognizes cancer cells expressing the folate receptor. pH-sensitive: cyt *c* is released at pH 4.0. Teranostic potential: the dye assesses the progress of cyt *c*-induced apoptosis	No	(Not estimated)	Lung and breast cancer	A549 and MCF-7 cells

**Figure 3:**
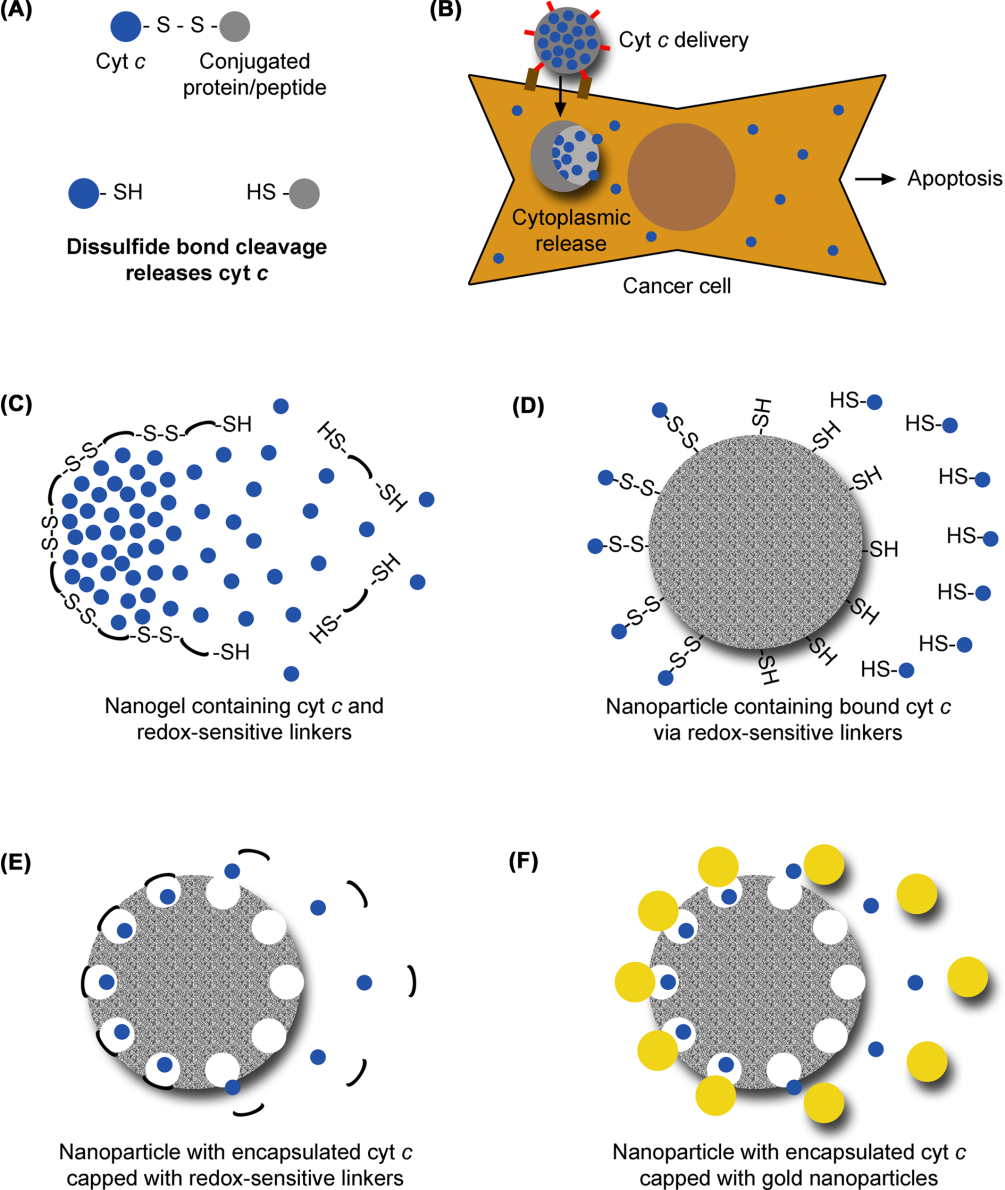
Systems for therapeutic delivery of cyt *c* to cancer cells (**A**) In fusion protein-based delivery systems, cyt *c* (represented in blue) is connected to its conjugated protein or peptide via a linker containing a disulfide bond. In the cytoplasm of cancer cells, the reductive chemical environment induces the cleavage of the disulfide bond and consequent release of cyt *c*. (**B**) Nanoparticle-based delivery systems are usually coated with ligands (represented in red) that recognize cancer cells through surface makers (represented in brown). In the cytoplasm, they release cyt *c*, which will induce the apoptosis of the cancer cell. (**C–F**) Nanoparticle-based delivery systems. In these systems, cyt *c* is encapsulated inside the nanoparticle or bound to it (left side of each drawing). In the cytoplasm, cyt *c* is released (right side of each drawing). (**C**) Cyt *c* can be encapsulated inside a nanogel containing redox-sensitive linkers (represented in black), whose disulfide bonds are cleaved in the cytoplasm of cancer cells, releasing cyt *c*. (**D**) Cyt *c* can be bound to nanoparticles via redox-sensitive linkers, whose disulfide bonds are cleaved inside the cytoplasm of cancer cells, releasing cyt *c*. (**E**) Alternatively, cyt *c* can be encapsulated inside enlarged nanoparticle pores caped with redox-sensitive linkers (represented in black), whose disulfide bonds (not represented in the figure) are cleaved in the cytoplasm, releasing cyt *c*. (**F**) In the system described in (**E**), nanoparticles containing cyt *c* can alternatively be capped with gold nanoparticles (represented in yellow) that are displaced by the electrical charge reversal caused by low pH. Elements were not drawn to scale. For simplicity, some of the elements of these delivery systems were omitted.

The protein-based cyt *c* delivery systems consist of cyt *c* fused to a specific protein or peptide. The fused protein or peptide is the ligand for a receptor that is expressed on the surface of specific cancer cells [47–50]. Some of these systems contain a disulfide bond that is cleaved in the cytoplasm [48,50], due to its reductive chemical environment, releasing cyt *c* (Figure 3A and Table 2). Released cyt *c* triggers apoptosis of the cell. In one of these systems, cyt *c* was fused to an antiepidermal growth factor receptor 2 (HER-2) antibody, for HER-2-positive breast cancer cell targeting [47]. In another system, cyt *c* was fused to transferrin, for targeting cancer types in which the transferrin receptor is overexpressed, including lung cancer [48]. For targeting glioma cells, cyt *c* was fused to the neurotoxin peptide chlorotoxin, to promote uptake into brain cells [49]. In another fusion system, cyt *c* was conjugated with galactosylated albumin, which is recognized by receptors on hepatocellular carcinoma cells [50].

The nanoparticle-based cyt *c* delivery systems are more diverse, including encapsulating pocket structures, nanoparticles with cyt *c*-loaded cavities and nanoparticles with cyt *c* in their own composition.

One of these groups consists in enclosing cyt *c* inside pockets formed by large molecular structures that can target cancer cells and release cyt *c* into the cytoplasm, inducing their apoptosis (Figure 3B). Those large structures where cyt *c* is encapsulated include ferritin (an iron-storage protein) [51], polypeptide micelles [52], and nanogels [53,54]. To ensure their specific targeting to cancer cells, some of these pocket structures were modified on their surface with ligands that bind specific molecules on the surface of cancer cells, triggering their endocytosis (Table 3). The polypeptide micelle delivery system is sensitive to oxygen scarcity within tumors, releasing its content faster under hypoxic conditions [52]. Additional nanoparticle-based systems are nanogels sensitive to the reductive chemical environment in the cytoplasm of cancer cells [53,54]. Reductive environment causes the reduction in disulfide bonds in the nanogels and their consequent disruption, which releases cyt *c* (Figure 3C).

In other nanoparticle-based systems, cyt *c* is part of the nanoparticle composition (Table 4). In some of these systems, cyt *c* is co-precipitated with a desolvating reactant to form the nanoparticles [55–57]. These nanoparticles also contain a cross-linking polymer with a disulfide bond. The disulfide bond is cleaved under reductive environment, releasing cyt *c* into the cytoplasm. In other systems (Table 4), cyt *c* is associated to lipid particles, in the absence [58] or presence of apolipoprotein A1 [59]. The lipid particle systems are generally not sensitive to the redox status of the cytoplasm, and take advantage of the lipid cell permeability to reach the cytoplasm.

Other delivery systems utilize a different type of nanoparticles (Table 5). In one of these systems, cyt *c* is chemically bound to the surface of nanoparticles composed of iron oxide and gold [60]. In another system, cyt *c* is chemically bound to the surface of mesoporous silica nanoparticles via a reducible disulfide bond [61]. This bond is cleaved under reductive environment and cyt *c* is released (Figure 3D). In other mesoporous silica nanoparticle delivery systems, the pores are enlarged to accommodate cyt *c* molecules. These cavities are further sealed with linkers containing disulfide bonds [62]. Reductive chemical environment in the cell cleaves the linkers, leaving the pores open to release cyt *c* (Figure 3E). Other versions of this system utilize electrostatic interactions to bind cyt *c* to the cavities, being sensitive to pH. Here, positively charged cyt *c* is bound to the negatively charged particles via electrostatic interactions. The acidic pH in cancer cells inverts the electrical charge of the nanoparticles, causing the disruption of electrostatic interactions and consequent release of cyt *c* [63–65]. In one of these systems, cyt *c*-loaded pores are capped with smaller gold nanoparticles bound through electrostatic interactions. Acidic environment inverts electrical charge causing the detachment of the gold nanoparticles and the release of cyt *c* [63] (Figure 3F).

These delivery systems are composed of biocompatible materials, which are effective in avoiding immune reactions of the host. Immune reactions against a cyt *c* delivery system would trigger its destruction and compromise its therapeutic effectiveness. To further ensure its biocompatibility in human cells, human cyt *c* should be utilized in the preparation of its delivery systems.

## Impact of cyt *c* mutations, post-translational modifications, and redox state in apoptosis

While the delivery of cyt *c* to cancer cells demonstrates to be a promising therapeutic strategy, attention should also be paid to the effectiveness of cyt *c* interaction with Apaf-1 [7], a critical step in the triggering of apoptosis. Lysine mutants of cyt *c* have shown deficient apoptosome function, by inhibiting Apaf-1 oligomerization [66]. On the other hand, three cyt *c* pathogenic variants, Gly41Ser, Tyr41His, and Ala51Val, could increase the efficiency of apoptosome activation, possibly by enhancing cyt *c* flexibility and facilitating its interaction with Apaf-1 [67].

An extensive set of post-translational modifications has been described in cyt *c* [68]. The proapoptotic capacity of cyt *c* is also dependent on its phosphorylation state. Cyt *c* phosphorylations inhibit its apoptotic functions, suggesting a cytoprotective role [69]. Mimicking phosphorylation of cyt *c* at serine 47 had a protective effect against apoptosis in the brain [70]. Cyt *c* phosphorylation at threonine 58 inhibited apoptosis in rat kidney [71]. In addition, the phosphorylation of cyt *c* at tyrosines 48 or 97 has been shown to decrease its proapoptotic activity. Cyt *c* point mutants mimicking the phosphorylation of tyrosine 48 [72] or tyrosine 97 [73] have shown decreases in caspase-3 activation of approximately 60% and 26%, respectively.

Other cyt *c* modifications have also shown antiapoptotic effects. Cyt *c* acetylation at lysine 53 promoted apoptosis evasion in prostate cancer xenografts [74]. Cyt *c* nitrations at tyrosines 46 and 48 have impaired the formation of functional apoptosomes [75] and might even promote cyt *c* degradation [76]. The nitration of tyrosine 74 was shown to block the activation of caspase-9, a critical step in the induction of apoptosis [77]. On the other hand, lysine N-homocysteinylation [78] could be proapoptotic. This modification has been related to increased cyt *c* peroxidase activity, which is associated with its efflux into the cytoplasm [79] and subsequent apoptosis triggering.

Moreover, only the oxidized (but not the reduced) form of cyt *c* could induce caspase activation and apoptosis [80]. In agreement with the inhibition of apoptosis in cancer, cyt *c* became excessively reduced in human brain and breast cancers, in comparison with healthy tissue [81].

From the above, the cyt *c* version to be used in the preparation of therapeutic delivery systems should be carefully chosen. While specific point mutations and the oxidized state of cyt *c* can enhance its apoptotic function, several post-translational modifications may compromise it and should be avoided.

## Approaches for enhancing cyt *c* thermal stability

A critical limitation of protein-based therapeutic delivery systems is the thermal stability of the protein, which limits its actual potential. Unfortunately, in low-income countries, which frequently have warm climates, it is still challenging to keep medicines in permanent refrigerated conditions from production to patient administration [82]. This is a frequently overlooked issue. The nanoparticle-based cyt *c* therapeutic delivery systems are likely to enhance the stability of the protein in comparison with bare cyt *c*. Nevertheless, additional strategies have been developed to further increase cyt *c* thermal stability, including PEGylation, ionic liquids, and B-DNA.

PEGylation consists in the chemical modification of a molecule with PEG, a polymer composed of ethylene glycol monomers. Cyt *c* was PEGylated with 5 kDa PEG polymers attached to lysine amino acids. This modification did not cause any major alterations in the protein’s secondary or tertiary structures. Using this modification procedure, each cyt *c* molecule was bound to either four or eight PEG molecules. Peroxidase activity was measured as an assessment of cyt *c* stability. Cyt *c* molecules with eight PEG molecules bound were the most stable, retaining 30–40% more peroxidase activity over 60 days (stored both at 4 and 25°C) than unmodified cyt *c*. Unmodified cyt *c* had a half-life of about 44 days at 4°C and about 24 days at 25°C, while its modification with either four or eight PEG molecules extended its half-life to at least 60 days at both temperatures. After 60 days of storage at 4°C, cyt *c* modified with four or eight PEG molecules retained 62% and 80% of their initial peroxidase activity, respectively; while unmodified cyt *c* showed only 43% of its initial activity. At 25°C, unmodified cyt *c* had still 27% peroxidase activity, in contrast with at least 53% of both PEGylated forms stored at the same temperature [83]. PEGylation has been shown to enhance the thermal stability of cyt *c*, not only in solution but also as a biosensor component. In a cyt *c*-based biosensor, cyt *c* was modified with 2 kDa PEG molecules and remained electrochemically active after a 2-month storage at 40°C [84].

For optimal stability increase, PEG molecules should be chemically bound to specific amino acids of cyt *c*, instead of being only added to the protein. Unspecific interactions may not always have a protective effect on the protein stability. The unspecific interaction of cyt *c* with 10 kDa PEG perturbed cyt *c* structure, while 20 kDa PEG had a protective effect. The protective effect of the 20 kDa PEG polymer was attained by excluded volume effect, which limited the interactions of cyt *c* with potentially denaturing molecules [85]. However, unspecific interaction of cyt *c* with high concentrations of 4 kDa PEG perturbed the secondary and tertiary structures of the protein [86]. Nevertheless, the interaction between cyt *c* and ethylene glycol monomers contributed to its stabilization [87]. PEGylation with an excessive number of PEG molecules per cyt *c* molecule should also be avoided. Specific binding of 0.4 kDa PEG at high concentrations affected the tertiary structure of cyt *c* and exposed hydrophobic regions [88].

In addition to PEGylation, ionic liquids have also been shown great value in extending cyt *c* thermal stability and shelf life. Although ionic compounds are generally solid, some of them have melting points under 100°C. These ionic liquids are salts in which the positive ion is bulky and organic and the negative ion is organic or inorganic. Their physicochemical properties can be modulated through the careful choice of the positive and/or negative ion(s), and their modifications. Ionic liquids have demonstrated the ability to stabilize proteins during production, transport, and storage, making them of outstanding value in the utilization of proteins as therapeutic tools [89]. Because of these properties, the interest around the potential of ionic liquids has grown considerably [90].

Ionic liquids based on cholinium dihydrogen phosphates tend to improve the thermal stability of proteins, an effect that strongly depends on their negative ion [89]. In aqueous buffers, cyt *c* is stable only for 1 week, while in a buffered cholinium dihydrogen phosphate solution, its shelf life can be extended for up to 6 months [91]. Cyt *c* was cryopreserved in a solution containing ethylammonium nitrate and 1-butyl-3-methylimidazolium thiocyanate ionic liquids. Although this solution caused cyt *c* denaturation, at least 90% of cyt *c* structure and function were recovered after its removal from the protein [92]. Cyt *c* stability and activity were also improved through ionic liquids based on amino acids [93]. Stability and conformational dynamics of cyt *c* could be modulated by using ammonium ionic liquids of different alkyl chain lengths [94]. Ionic liquids based on amino acids were also tested as solvents for storage of cyt *c*. These liquids do not alter the structure of cyt *c* [93]. The long-chain imidazolium ionic liquids 1-methyl-3-octyl imidazolium chloride and 1-decyl-3-methylimidazolium chloride at less that 1 mM were shown to increase cyt *c* stability during its long-term storage at room temperature for 6 months [95]. Another ionic liquid, cholinium glutarate, was also successful in extending cyt *c* stability and catalytic activity. Storage at room temperature could be extended to 5 months. Cyt *c* could also be recovered from this ionic liquid without compromising its properties [96]. Packaging of cyt *c* within gold nanoparticles coated with ionic liquid salts increased cyt *c* activity and extended its thermal stability and long-term storage [97].

Concerning cyt *c* thermal stability, it is also useful to know the properties that lower its thermal stability. Among ionic liquids, water-diluted salts containing the imidazolium cations 1-ethyl-3-methylimidazolium or 1-butyl-3-methylimidazolium could strongly destabilize cyt *c* thermal (but not pH) stability, especially the butyl salt. They destabilize through binding and by decreasing surface tension of the solvent [98]. There are some concerns regarding toxicity of ionic liquids; nanoparticles appear to be safer. Their conjunction holds valuable promise in improving drug stability, and consequently, their performance, as the ionic liquids can also improve thermal and chemical stability of nanoparticles [99]. On the other hand, PEGs are considered remarkably safe, a quality that makes them frequently used in cosmetic products [100]. Nevertheless, it should be kept in mind that PEGylation of cyt *c* may affect its interaction with Apaf-1, which is a critical step in apoptosome formation. As such, the compatibility of cyt *c* PEGylation with its anticancer exploitation should be carefully assessed.

Besides PEGylation and ionic liquids, B-DNA (the most commonly found DNA conformation) is also a potentially useful tool to improve cyt *c* stability. Packaging of cyt *c* inside B-DNA scaffolds increased its peroxidase activity. Its stability under high temperature, high pH, oxidative stress, and denaturants was also enhanced [101]. On the other hand, electrostatic adsorption of cyt *c* on DNA induced self-aggregation of the protein [102]. Therefore, DNA as a cyt *c* stabilizing agent also needs to be carefully used.

These approaches are promising for extending the shelf life of cyt *c* under limited refrigeration conditions. Regarding protein preparation, cyt *c* is usually purified from recombinant bacterial expression systems [103], providing high yield and purity. In addition, novel and highly selective approaches for cyt *c* purification from mammalian extracts have been developed [104,105]. The conjunction of highly selective cyt *c* purification with better preservation at room temperature has the potential of extending usability of cyt *c* therapeutic delivery systems in settings with limited refrigeration.

## Discussion

The present review highlights the potential of cyt *c* in cancer. Monitoring of its levels in the serum can be informative of the patient’s response to chemotherapy (Figure 1), for which several highly sensitive biosensors have been developed (Figure 2 and Table 1). Moreover, cyt *c* can be a chemotherapeutic drug when delivered into cancer cells, for which several delivery systems have also been developed (Figure 3 and Tables 2–5). The therapeutic potential of cyt *c* under limited refrigeration conditions might be further enhanced through PEGylation and storage in ionic liquid solutions, which enhance its thermal stability.

The biosensors for quantification of cyt *c* in the serum demonstrate the versatility of the methodologies available to detect this protein in biological samples. Additional biosensors have been validated in solution, cell lysates, or even for live-cell visualization. These biosensors could also be tested for quantification of cyt *c* in human serum. Unlike in cancer, cyt *c* is elevated in the serum of myocardial infarction patients [106], for which some cyt *c* biosensors have also been developed [21,22]. High levels of cyt *c* in the serum has also been detected in HIV patients receiving antiretroviral therapy, indicating cyt *c* as a marker of antiretroviral toxicity in HIV patients [107]. Cyt *c* mitochondrial efflux has also been reported in neurodegenerative diseases including Parkinson’s disease [108] and Huntington’s disease [109], in which extensive apoptosis of neuronal cells in implied [110]. However, cyt *c* fails to cross the blood–brain barrier [111], which limits the possibility of quantifying cyt *c* released from neuronal cells. Nevertheless, these observations suggest that quantification of cyt *c* in patient serum could also be useful to monitor the response to treatment and the prognosis of other diseases in which apoptosis is inhibited or exacerbated.

Concerning the cyt *c* therapeutic delivery systems, many of them are equipped with ligands of proteins that are specifically expressed on cancer cells, to avoid cytotoxic effects in healthy tissue. This is an advantage over chemotherapeutic drugs that induce more systemic apoptosis, with severe effects to the patient. Although protein delivery into cells is a valuable therapeutic approach, its production inside patient cells emerges as an attractive alternative. Gene therapy has been successful in delivering therapeutic genes to the patient, for *in vivo* production of therapeutic proteins. Of note, cancer types including melanoma lesions have been treated with gene therapy [112]. Unfortunately, a gene therapy approach might not be effective for increasing cyt *c* levels in the cytoplasm. After its translation, cyt *c* is extensively imported into mitochondria. Its folding and heme attachment take place in the mitochondrial intermembrane space [113]. Therefore, the delivery of a recombinant gene coding for cyt *c* into patient cells may result in increased cyt *c* levels in mitochondria and not in the cytoplasm.

In addition to developing innovative therapeutic tools, providing their wide availability to patients should also be a concern. This includes patients from low-income countries, whose clinical facilities may have limitations in refrigeration equipment. The strategies to enhance the thermal stability of cyt *c*, such as PEGylation and storage in ionic liquid solutions, should now be tested in cyt *c* therapeutic delivery systems. Importantly, cyt *c* PEGylation may block its binding to Apaf-1 and subsequent apoptosome formation. Therefore, straightforward methods for the removal of PEG molecules from cyt *c* immediately before patient administration could be needed. Similar strategies for the removal of ionic liquids used in cyt *c* storage could also be needed, due to the safety concerns associated with these solvents. If successfully tested, these approaches should extend the room temperature shelf life of cyt *c* therapeutic delivery systems, making them more available to patients in clinical facilities with limited refrigeration conditions.

In conclusion, sensitive quantification of cyt *c* in patient serum emerges as a noninvasive prognosis biomarker to assess the effectiveness of cancer therapy. Moreover, the delivery of cyt *c* to cancer cells has shown a promising therapeutic potential. The cyt *c* therapeutic delivery systems may become more widely available if their thermal stability could be extended.

## Abbreviations

Apaf-1: apoptotic protease activating factor-1
Cyt *c*: cytochrome *c*
ELISA: enzyme-linked immunosorbent assay
HER-2: epidermal growth factor receptor 2
PEG: polyethylene glycol
SERS: surface-enhanced Raman scattering
